# Promoting interdisciplinarity and the timely integration of palliative care through the development and implementation of a blended learning elective for medical students

**DOI:** 10.3205/zma001748

**Published:** 2025-04-15

**Authors:** Yann-Nicolas Batzler, Manuela Schallenburger, Tabea Sammer, Jan Haussmann, Bálint Tamaskovics, Marc Rehlinghaus, Julia von Schreitter, Stefanie Otten, Corinna Fohler, Jacqueline Schwartz, André Karger, Günter Niegisch, Martin Neukirchen

**Affiliations:** 1 Heinrich-Heine-University Düsseldorf, Medical Faculty and University Hospital Düsseldorf, Interdisciplinary Centre for Palliative Medicine, Düsseldorf, Germany; 2 CIO ABCD (Aaachen-Bonn-Cologne-Düsseldorf), Centre for integrated oncology (CIO) Düsseldorf, Düsseldorf, Germany; 3 Heinrich-Heine-University Duesseldorf, Medical Faculty and University Hospital Düsseldorf, Department of Radiation Oncology, Duesseldorf, Germany; 4 CIO ABCD (Aaachen-Bonn-Cologne-Düsseldorf), Centre for integrated oncology (CIO) Düsseldorf, Duesseldorf, Germany; 5 Heinrich-Heine-University Düsseldorf, Medical Faculty and University Hospital Düsseldorf, Department of Urology, Düsseldorf, Germany; 6 Heinrich-Heine-University Düsseldorf, Medical Faculty and University Hospital Düsseldorf, Institute for Psychosomatic Medicine and Psychotherapy, Düsseldorf, Germany; 7 Heinrich-Heine-University Düsseldorf, Medical Faculty and University Hospital Düsseldorf, CoMeD, Düsseldorf, Germany; 8 Heinrich-Heine-University Düsseldorf, Medical Faculty and University Hospital Düsseldorf, Department of Anesthesiology, Düsseldorf, Germany

**Keywords:** palliative care, medical education, health promotion, timely integration, learning gains, elective

## Abstract

**Background:**

Given demographic changes and a rising prevalence of oncological diseases, understanding the importance of interdisciplinary collaboration and a timely integration of palliative care is crucial. However, both are underrepresented in medical curricula. To address this gap, we introduced a new elective in which students follow the journey of a fictitious patient with prostate cancer from diagnosis until death.

**Method:**

The elective was conducted through repeated joint meetings by a multi-professional and interdisciplinary (palliative care, urology, radiation oncology, psychosomatic medicine) team. Alongside its development, an outcome evaluation was designed to assess satisfaction (Likert scale) and learning gains (comparative self-assessment, CSA [%]). After pilot testing, the content and structure were adapted. The elective followed a blended learning approach. The content covered guideline-adherent treatment of prostate cancer, breaking bad news, initial contact with palliative care, symptom control based on the total pain concept.

**Results:**

Students (n=8) expressed high satisfaction. They found the structure comprehensible and considered the content valuable for medical practice. Students gained knowledge, especially in defining total pain (83%) and the indication of the timely integration of specialized palliative care (77%).

**Conclusion:**

Using prostate cancer as an example disease, we integrated multiple disciplines into treatment strategies, demonstrating the benefits of multi-professional and multidisciplinary collaboration. This approach aids in identifying patients who could benefit from palliative care. Our concept is adaptable to other tumor types and settings, enhancing awareness of patient-centered issues that are often overlooked in medical curricula.

## Zusammenfassung

**Hintergrund:**

Angesichts des demographischen Wandels und einer steigenden Prävalenz onkologischer Erkrankungen sind interdisziplinäre Zusammenarbeit und eine zeitgerechte Integration der Palliativmedizin essenziell. Beides ist in medizinischen Lehrcurricula unterrepräsentiert. Daher wurde ein Wahlpflichtfach entwickelt, in dem Student:innen einen Patienten mit Prostatakarzinom von Erstdiagnose bis zum Versterben begleiten.

**Methodik:**

Das Wahlpflichtfach wurde durch ein multiprofessionelles und interdisziplinäres Team bestehend aus Lehrenden der Palliativmedizin, Urologie, Strahlentherapie und psychosomatischen Medizin entwickelt. Parallel wurde eine Evaluation konzipiert, die die Zufriedenheit und Akzeptanz (Likert-Skala) sowie den Lernzuwachs (CSA-Gains (%)) erfassen sollte. Nach einer Pilotierung wurden Inhalte und Struktur angepasst. Das Wahlfach folgte einem Blended-Learning-Ansatz. Die Inhalte umfassten die leitliniengerechte Therapie des Prostatakarzinoms, Überbringen schlechter Nachrichten, Erstkontakt mit der Palliativmedizin sowie Symptomkontrolle auf Basis des Total Pain Konzepts.

**Ergebnisse:**

Die Student:innen (n=8) äußerten eine hohe Zufriedenheit. Sie bewerteten die Struktur des Wahlpflichtfaches als nachvollziehbar und die Inhalte als wertvoll für die medizinische Praxis. Besonders ausgeprägt war der Wissenszuwachs zum Total Pain Konzept (83%) sowie zur zeitgerechten Integration der Palliativmedizin (77%).

**Schlussfolgerung:**

Anhand der Erkrankungsentität „Prostatakarzinom“ konnten verschiedene Disziplinen in Behandlungskonzepte integriert und die Vorteile interdisziplinärer Zusammenarbeit aufgezeigt werden. Das Stärken kooperativer Fähigkeiten im klinischen Alltag kann die Identifikation von Patient:innen, die von Palliativmedizin profitieren, erleichtern. Unser Konzept ist auf andere Tumorarten und Kontexte übertragbar und erhöht das Bewusstsein für patientenzentrierte Themen, die in medizinischen Curricula häufig vernachlässigt werden.

## Introduction

Guidelines from different medical fields, such as gynecology, urology, and intensive care medicine [1], [2], [3], implemented the timely integration of palliative care in patients’ disease trajectories, furthermore, interdisciplinarity is demanded. Recently, a shift was propagated away from inclusion of palliative care at the final stages of diseases towards a collaborative approach at earlier disease stages [4], [5], [6], [7], [8]. Palliative care treats patients on four dimensions: physical, psychological, social, and spiritual. This concept dates back to Dame Cicely Saunders in the 1960s, who emphasized that suffering is not a unidimensional phenomenon but manifests across multiple domains, including the aforementioned aspects [9], [10], [11]. This multifaceted understanding of suffering is encapsulated in the term “total pain”. Consequently, the holistic treatment of patients should address symptoms across all these dimensions. To achieve this, treatment strategies must integrate the expertise of various professional disciplines, including nurses, physicians, pastoral care providers, social workers, and psycho(onco)logists, among others.

Many studies identified benefits for both patients and their families when integrating palliative care: the quality of life of patients can improve, next of kin are comforted, and patients’ lives might even be prolonged [12], [13]. However, a timely integration is often omitted by both patients and health care professionals [14], [15], [16]. Putting a focus on interdisciplinarity could be a way to enhance treatment strategies for patients. Among the general population, palliative care faces stigmatization as it’s being associated with death, hopelessness, and social exclusion [14], [17], [18], [19]. Healthcare professionals often fear that integrating palliative care may instill a sense of hopelessness in patients and perceive it as a personal failure or an admission of giving up [20], [21], [22], [23], [24], [25], [26].

Only those healthcare professionals who realize the benefits of ongoing alliances in favor of the patients’ overall health and quality of life will be more likely to cooperate with other disciplines or professions [27]. In order to facilitate the integration of other medical specialties, like palliative care, into patients’ disease trajectories, interdisciplinary and multi-professional collaboration is needed [28]. Furthermore, as a way to promote a much-demanded timely integration of palliative care and collaborating in an interdisciplinary manner, a shift in perception and knowledge has to take place among medical professionals. In public health, younger population strata are of interest in public campaigns and interventions. Since their health behavior can still be altered and influenced, they are a crucial target population [14]. It is therefore crucial to emphasize education on palliative care within medical curricula to foster a positive attitude toward palliative care and promote interdisciplinary collaboration among future physicians. 

In Germany, palliative care education was formally integrated into medical curricula as a compulsory subject in 2013. However, its implementation remains heterogeneous: while some medical faculties offer a combination of seminars, lectures, and bedside teaching, others focus solely on lectures. In order to complement compulsory teaching, many German medical faculties offer electives on different medical disciplines and topics to deepen students’ knowledge. To develop specific skills and interact with interested medical students on a deeper level, we offer four electives centered around palliative care at our facility: handling the desire to die [29], communication in critical situations [30], voluntary hospice service, and interprofessional palliative care. While this range of courses offer a multitude of relevant topics surrounding palliative care, none focus specifically on interdisciplinary collaboration and timely integration of palliative care. With the development of a new elective, we aimed to fill this gap. 

It is the aim of this work to present the structure of a new elective focused on the timely integration of palliative care and the importance of interdisciplinary collaboration. Furthermore, results of an evaluation among participating students are presented which assess the impact of the elective in terms of knowledge acquisition, acceptance, satisfaction, and the development of crucial clinical and collaborative skills.

## Methods

This study is a single-center prospective study conducted at University Hospital Düsseldorf, Germany. Ethical approval was obtained by the local ethics committee (reference number 2023-2652).

### Development of the elective

The elective was developed over the course of one year through repeated interprofessional and interdisciplinary discussions and meetings. The development was funded by the state of North Rhine-Westphalia (“Qualitätsverbesserungsmittel”). The elective was named “jungle oncology” to reflect the forlornness many oncological patients experience during their treatment journeys. 

The development team included physicians, nurses and didactics experts. The medical disciplines involved were urology, radiation oncology, psychosomatic medicine, and palliative care. The development followed the Kern-cycle [31]. Following the *identification of problems* and a *needs assessment*, the *goals* of the elective were established: to enhance knowledge of prostate cancer treatment, to understand the importance of interdisciplinary collaboration, and to learn how to integrate palliative care at an appropriate time. Learning methods (detailed below) were selected, and an evaluation plan (outlined below) was developed. The content was based on the requirements of the German medical curricula, as agreed upon by German medical faculties [https://nklm.de/zend/menu], as well as guidelines (prostate cancer, palliative care), existing relevant literature [2], [32], [33] and personal experiences. Good practice videos were produced in advance with the help of the “Multimediazentrum”, a joint venture of University Hospital Düsseldorf and Heinrich-Heine-University Düsseldorf. Participating actors are members of Heinrich-Heine-University’s simulated patients program “CoMeD” (Communication in Medical Education Düsseldorf), certified by the German Association of Medical Education. The elective was carried out during the course of one semester with four students participating in a pilot phase. Based on students’ evaluations and feedback from the study team, the content was adapted, including adjustments to structure and the addition of new information. From these experiences, it was determined that the elective would be limited to a maximum of eight students (minimum: four) per semester to maintain a private and focused learning environment.

### Structure of the novel elective and content taught

Throughout the elective, students follow the journey of a fictitious patient with prostate cancer, progressing from curative care to best supportive care (see “case description”). Prostate cancer was chosen as an ideal disease for this elective because it requires collaboration among multiple disciplines and involves various targeted therapies from which patients can choose. Additionally, its usually relatively slow progression and overall good 5-year-survival rates allow for the discussion of timely palliative care integration at different disease stages. 

**Figure 1:**
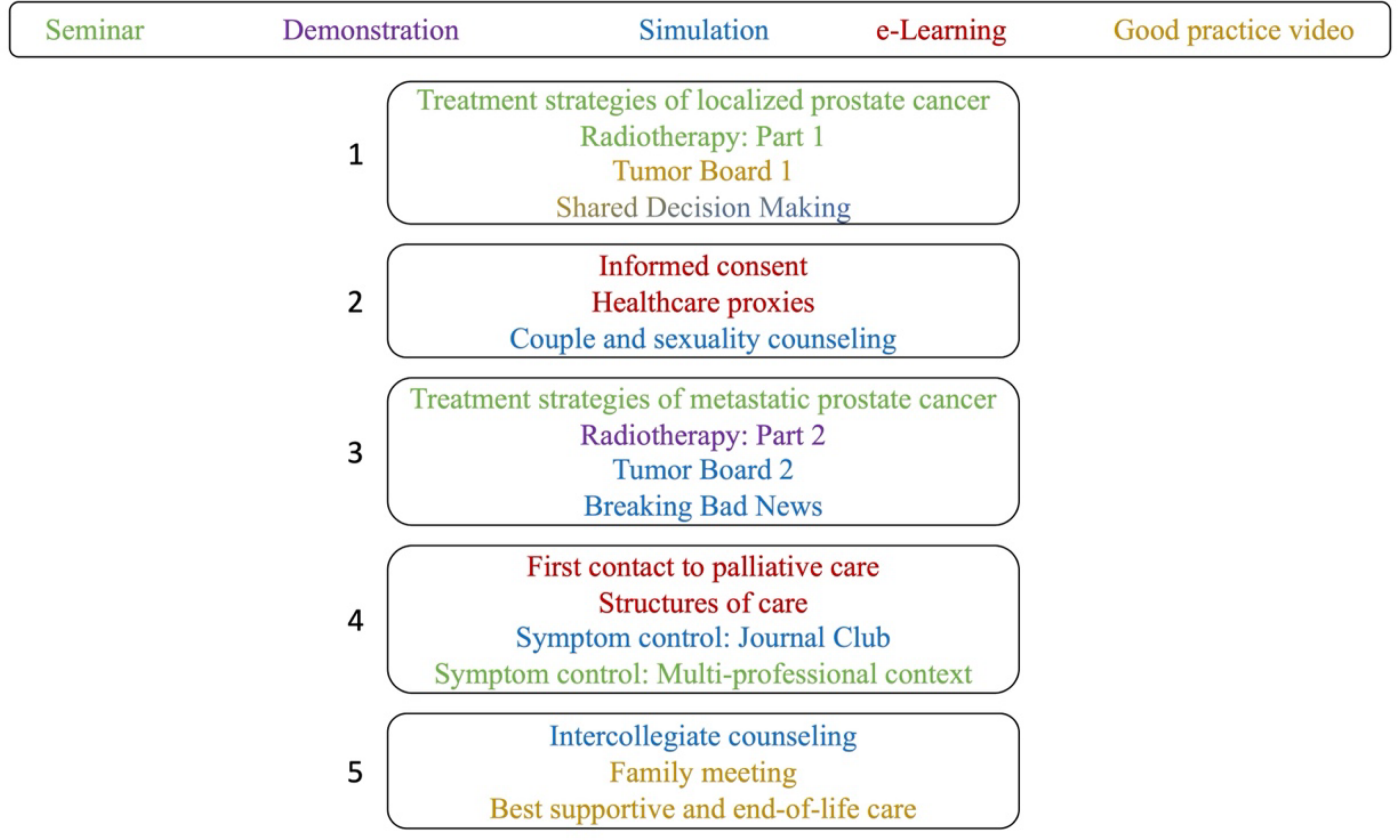
The elective is made up of five units and makes use of a blended learning approach (*Shared decision making* was taught through both a good practice video and a simulation)

The elective was structured into five units, each comprising eight hours. It employed a blended learning approach that included e-learning, live seminars, live demonstrations, group simulations with simulated patients, and good practice videos produced for this course. The topics covered were breaking bad news, couple counselling (sexual dysfunction), shared decision-making, guideline-oriented treatment strategies for prostate cancer, the transition to palliative care, symptom control, and end-of-life care. These were taught by physicians and nurses from palliative care, urology, radio oncology, psycho-oncology and psychosomatic medicine. Figure 1 (Fig. 1) provides a summary of each unit, the content taught, and the learning formats used. *Treatment strategies of prostate cancer* was taught by urologists, *radiotherapeutic treatment strategies* as well as a *live demonstration of radiation treatment (“radiotherapy: part 2”)* by radiooncologists, *breaking bad news* and *couple counselling* by experts in psychosomatic medicine, *symptom control* and *end-of-life care* by palliative care physiscians, to name a few examples. The importance of interdisciplinarity was taught through the collaboration between urologists, radiooncologists and palliative care experts (seminars on treatment of prostate cancer and symptom control). This collaboration was highlighted in live seminars *(treatment strategies of prostate cancer)* and simulations *(breaking bad news)*. The timely integration of palliative care was taught in an e-learning session* (first contact to palliative care)*, good practice video *(family meeting)*, and simulations *(tumor board 2)*. 

**Figure 2:**
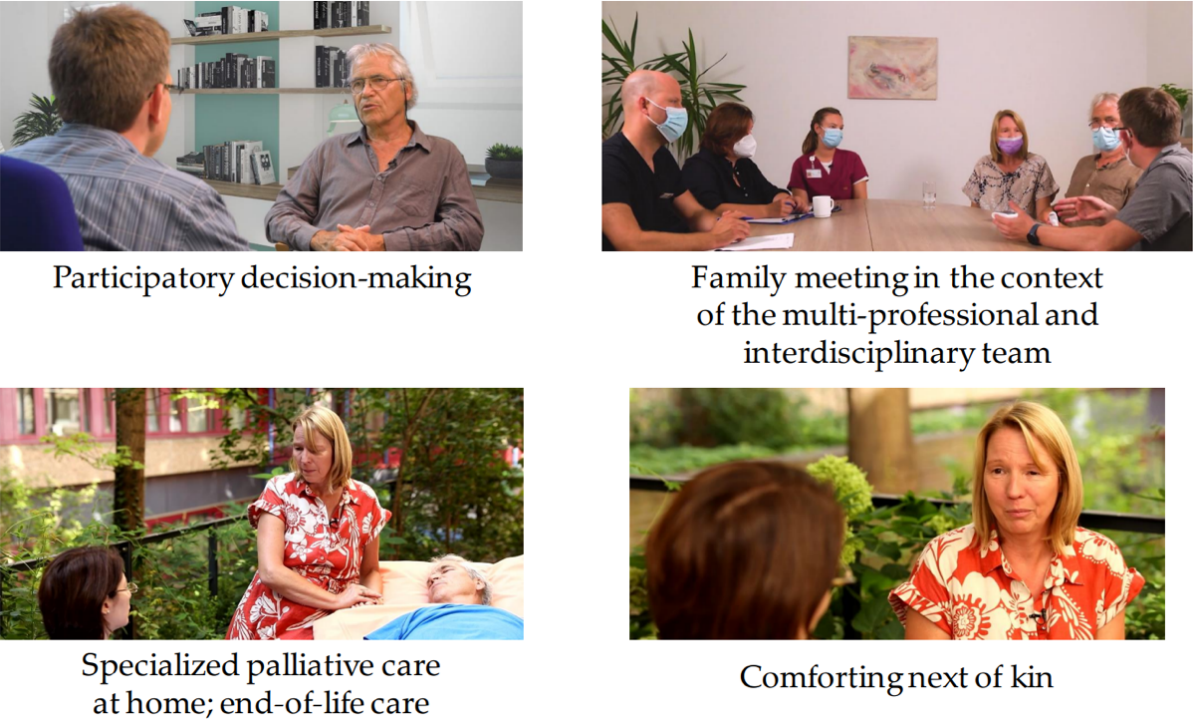
Content taught in good practice videos

Scenes from the good practice videos are shown in figure 2 (Fig. 2).

#### Case description

*Mr. Pollmann is a 57-year-old patient with no prior medical history. He has a wife, a daughter, and a granddaughter. He owns and operates a painting business and is known for being social and outgoing. During routine cancer screening, he is diagnosed with localized prostate cancer. After consulting with his primary urologist, he chooses to undergo prostatectomy instead of primary radiotherapy. Postoperatively, he experiences erectile dysfunction, which eventually resolves. Three years later, his prostate-specific antigen (PSA) levels rise, leading to salvage radiotherapy. After five years, his PSA levels increase again, and the first bone metastasis is detected. This prompts his main caregiver urologist to refer him to palliative care for the first time. Over the following years, Mr. Pollmann undergoes chemotherapy and multiple hormonal deprivation therapies. Despite these treatments, he develops several bone metastases. Due to severe pain, he is admitted to a palliative care ward. Seven months after his discharge, Mr. Pollmann passes away at home, supported by his wife, daughter, and an outpatient specialist palliative care team.*

### Evaluation

In literature, no validated instrument that met the needs for the evaluation of the novel elective was found. Therefore, a structured, paper-based questionnaire was developed in repeated interdisciplinary and multiprofessional discussions (see attachment 1 only in German ). The basis for the questionnaire were the learning goals that were defined during the development of the elective and the overall goals expressed in the German medical learning target index (“Nationaler Kompetenzbasierter Lernzielkatalog”) [2]. The questionnaire was pretested during the pilot phase among the four participating students. Since participating in the elective is a crucial determinant in being able to answer the questions, the questionnaire was not pretested among a larger cohort. Based on the experiences gained during the pilot phase, unclear questions were revised or removed.

Following the Kirkpatrick Model, levels 1 (“reaction”) and 2 (“learning”) were included in the outcome evaluation of the elective [34]. Level 3 (“behaviour”) was not measurable after completion of the elective since no follow-up was to take place. Level 2 was addressed on the knowledge, attitude, and skills plane.

The questionnaire consisted of two parts. The first part (11 items) focused on satisfaction and perception making use of answers on a five-point Likert scale (1: strongly disagree, 2: disagree, 3: neutral, 4: agree, 5: strongly agree). For a specific outcome evaluation, the second part of the questionnaire consisted of ten statements on the knowledge, skills, and attitude planes. Making use of the comparative self-assessment (CSA) method to determine if a gain in knowledge was achieved, each student evaluated their knowledge retrospectively before taking part in the elective and after attendance of the last unit of the elective using the German school grading system (1=“excellent” to 6=“unsatisfactory”) (post-then-design) [35]. 

The CSA gain is a well implemented method in evaluating acquisitions of knowledge and skills in education [35], [36]. This evaluation tool has the benefit of not taking into account experiences made beforehand as they are not contributing to the effect size. CSA gain is calculated as followed: CSA gain (%) = 
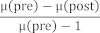
 x 100

### Participation and analysis

Participation in the study was anonymous, voluntary, and could be withdrawn at any time without providing a reason. Eligible participants were medical students aged ≥18 years who chose to enroll in the elective. A minimum attendance rate of 90% (4.5 units) was required to participate in the evaluation. The purpose and content of the study were presented orally at the beginning of the last unit, and written information and consent documents were distributed. Questionnaires were administered at the end of the last unit of the elective. There were no exclusion criteria other than refusal to participate or having missed more than four hours of the elective (equivalent to half a unit).

For the analysis of answers made on Likert scale, mean values were calculated. CSA gains were calculated with a 95% confidence interval and standard error using individual learning gain (ILG) values. These values were calculated using the following formulas:

- ILG = 0 if pre = post and
- ILG = (pre – post)/(pre – 1) × 100 if pre > post [36].

Data analysis was performed using Microsoft Excel 2023 for Mac (version 16.78, Microsoft Corp., Redmond, WA, USA) and JASP (version 0.18.3, Apple Silicon).

## Results

### Demographics

Over the course of one semester, eight students enrolled in the elective, including six females and two males. Four participants were in their third or fourth year of medical school (out of six total years in Germany), while the other four were in their fifth year. Five students were aged 22 to 23 at the time of participation, and the remaining three were 24 to 25 years old. All participating students took part in the study.

### Satisfaction and perception of the elective

**Figure 3:**
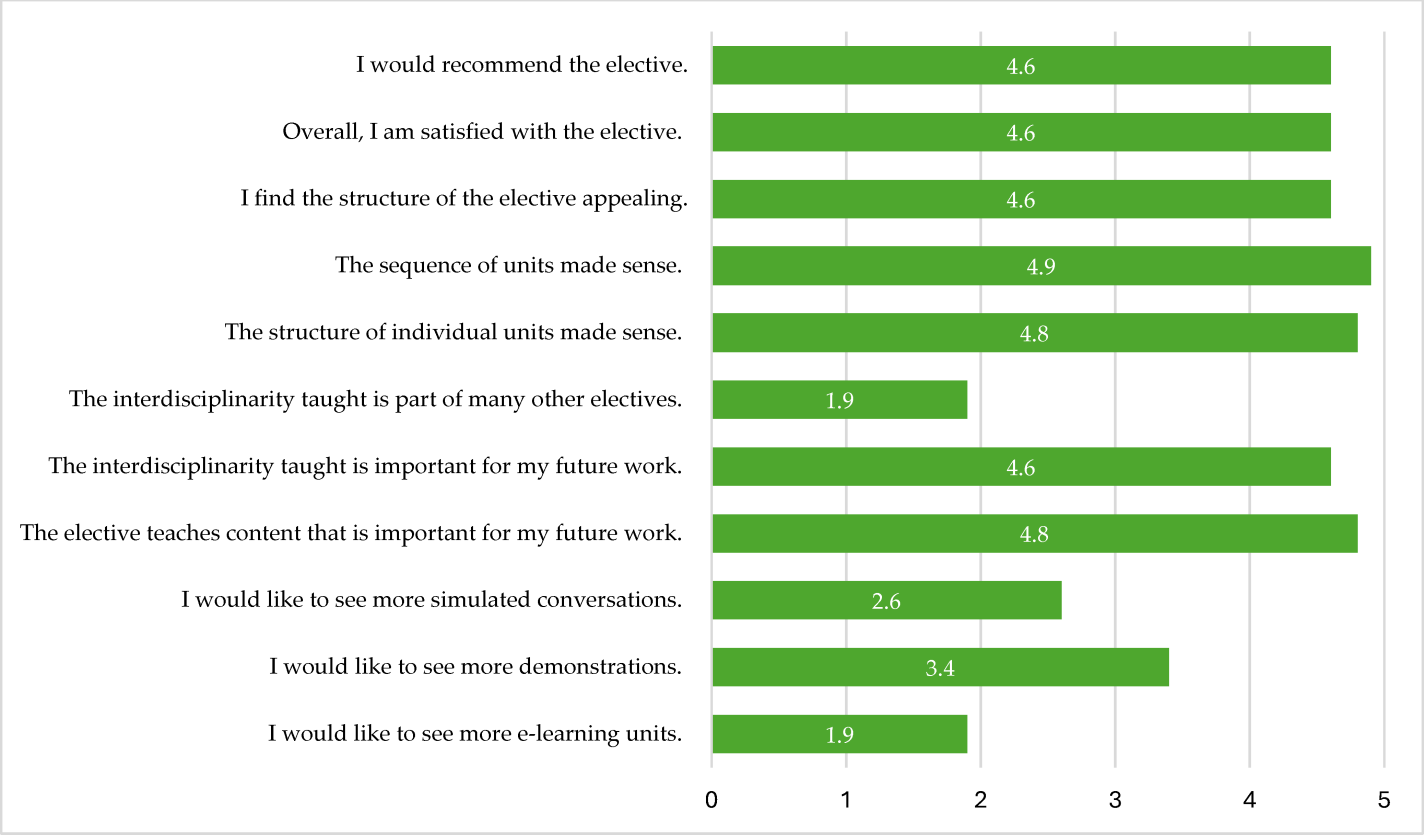
Evaluation, Likert scale, mean scores (1=strongly disagree, 2=disagree, 3=neutral, 4=agree, 5=strongly agree).

Overall, the students were highly satisfied with the elective (mean 4.6, min. 4, max. 5, SD 0.5) and would recommend it to other students (mean 4.6, min. 3, max. 5, SD 0.7). Participants did not attend other electives that emphasized on interdisciplinarity (mean 1.9, min. 1, max. 2, SD 0.4). When asked about relevance for their later clinical work, they perceived the elective to cover fields that are of importance (mean 4.8, min. 4, max. 5, SD 0.5). Further mean scores on Likert scale are shown in figure 3 (Fig. 3). 

### Specific outcome evaluation

The highest knowledge acquisition was observed in the ability to define the concept of “total pain” (item 7, gain: 83.3%). Students also reported an improved ability to determine the appropriate timing for referring patients to specialized palliative care (item 10, gain: 76.9%). On the attitude level, there was increased awareness of the importance of interdisciplinary collaboration after completing the elective (item 9, gain: 76.9%). The elective particularly focused on linking therapeutic strategies to communication skills. While participating students showed significant knowledge acquisition regarding treatment options for prostate cancer (item 1, 73.9%), their personal assessment of improvements in communication skills was not as pronounced (items 4 and 5). Overall, CSA gains were the highest on the knowledge level (mean: 72.2 %), followed by attitude (mean: 65.1 %), and skills (mean: 52.8 %). All results were significant as confirmed by confidence intervals. Table 1 (Tab. 1) and table 2 (Tab. 2) provide details for each item and corresponding CSA gains. 

## Discussion

The evaluation of the novel elective demonstrated overall positive acceptance of both its structure and content. It facilitated the acquisition of knowledge regarding the timely integration of palliative care and underscored the importance of continuous clinical collaboration. 

International guidelines mandate the timely integration of palliative care; however, healthcare professionals remain hesitant to refer patients to these services [20], [21], [22], [25], [26]. Efforts to promote a timely palliative care integration have traditionally focused on clinicians, with minimal attention given to medical students. In public health, young people are a crucial target population due to their malleable health behaviors [14]. However, reaching this demographic can be challenging. Our elective represents a novel approach, directly engaging young medical students and promoting interdisciplinarity and a timely integration of palliative care. Importantly, the elective also integrates other medical disciplines, emphasizing the collaborative nature in medicine. 

A study from the United States demonstrated that electives in palliative care can enhance the skills and knowledge of medical students, showing significant improvements in understanding pain and symptom management as well as advance care planning [37]. In our elective, prostate cancer served as an ideal disease model because it allows for the longitudinal study of a fictitious patient, facilitating the integration of various medical disciplines. Additionally, prostate cancer provides an opportunity to address sensitive issues such as patient sexuality. By leveraging the gradual transition from curative to palliative treatment, we were able to instruct students on the appropriate timing for referring patients to specialized palliative care. Outcome evaluations demonstrated that students learned about the indications for integrating palliative care. Additionally, they showed an improvement in their understanding of "total pain”, a fundamental concept in palliative care. On the other hand, the improvements in clinical skills, as measured by CSA gains, were less pronounced. However, similar trends were observed in other studies we conducted [29], [30], [38], [39], which is explicable: Competencies necessitate time for cultivation and are reinforced through clinical practice. Nevertheless, the elective may have enhanced their understanding and highlighted the critical importance of effective communication skills in routine clinical practice. To complement live communicative instructional sessions, we incorporated good-practice videos. Through these resources, participating students could observe potential role models and glean insights into the positive impacts effective communication can exert on patients. The small group size, blended learning and the thorough observance of one patient’s case might have contributed to relatively high gains on the attitude level since students engaged more personally with the content. 

A study conducted in the Netherlands demonstrated that medical electives can effectively highlight topics that are often underrepresented in standard medical curricula: A medical faculty's elective effectively imparted concepts and competencies in medical education to students, which will prove pivotal should they pursue employment at university hospitals where teaching responsibilities are mandatory [40]. Similarly, a study in the United States found that a palliative care elective impacted students at an attitudinal level by fostering empathy and self-reflection [41], both crucial attributes when engaging with patients navigating severe symptom crises or facing the end of life. In the context of our elective, we successfully integrated interdisciplinary teaching, thus impacting students' attitudes: They exhibited an enhanced appreciation for the significance of interdisciplinary collaboration and recognized its indispensability in their future clinical endeavors. Moreover, this aspect was perceived as neglected in comparison to other elective experiences. Beyond fostering an understanding of the merits of timely palliative care integration, our elective aimed at cultivating ongoing collaborations, recognizing their vital role in modern medicine by facilitating referrals across medical disciplines, thereby enhancing the quality of patient care [42].

Another aspect often insufficiently emphasized in German medical curricula pertains to post-discharge care structures. We aimed at addressing this deficiency by instructing students on these important structures. Students demonstrated a degree of confidence in articulating post-discharge facilities (such as rehabilitation centers, nursing homes, hospices) and home care structures (including nursing services and outpatient palliative care). Additionally, they acquired abilities to formulate discharge plans tailored to individual patients. Given the ongoing demographic shifts, competence in these skills will become increasingly essential in future clinical practice [43]. 

A study from Portugal identified agreement with teaching and learning methodologies as key determinants of student satisfaction in medical school electives [44]. To enhance comprehension and enjoyment, we adopted a blended learning format. Students expressed high satisfaction with the course content and did not request additional simulations or e-learning units. Our elective achieved high satisfaction levels with its structure and teaching methods, suggesting the success of our approach.

### Limitations

Our elective was conducted at a single institution, serving as an illustrative example rather than a definitive model. Evaluation results and specific outcomes may vary at other faculties. There is a potential for participation bias, as students with a strong interest in palliative care, multi-professionalism, and interdisciplinarity are more likely to enroll in the elective.

To facilitate deeper interactions and create a private, protected learning environment, the class size was limited to eight students per semester. This small sample size precludes broad generalizations, allowing only for the identification of trends. A qualitative approach in evaluating the elective could strengthen our results. 

## Conclusions

International medical guidelines mandate the timely integration of palliative care. Furthermore, interdisciplinary cooperation will become increasingly important. However, these topics have not been explicitly addressed in most medical curricula. Our novel elective aimed to bridge this gap. Utilizing a blended-learning approach (e-learning, seminars, demonstrations, simulations, good practice videos), the course covered numerous clinically important topics, especially interdisciplinary collaboration and a timely integration of palliative care, by following the disease and treatment trajectory of a fictitious prostate cancer patient. Participating students expressed high satisfaction with the structure and content of the elective and would recommend it to their peers. Students gained valuable knowledge about the importance of interdisciplinary collaboration, and the timely integration of palliative care. Our educational model is adaptable to other tumor types or clinical settings and helps raise awareness of critical patient-centered topics often overlooked in medical curricula. In the future, with the goal of placing greater emphasis on multi-professionalism, additional professions could be incorporated into the curriculum, providing insight into other domains such as art or aromatherapy. Furthermore, expanding the elective to include more than the eight students in this study should be considered, as medical cohorts often consist of up to a couple hundred students, allowing for a broader reach.

In order to validate the positive results in this pilot cohort, we recommend offering this elective at other medical faculties and conducting repeated outcome evaluations using validated instruments following a mixed-methods design. 

## Notes

### Author contributions

- Conceptualization: Y.-N.B., T.S., M.S., A.K., J.S., J.H., B.T., G.N., J.v.S., and M.N.
- Methodology: Y.-N.B. and M.S.
- Formal analysis: Y.-N.B.
- Resources: Y.-N.B.
- Writing – original draft preparation: Y.-N.B.
- Writing – review and editing: Y.-N.B., T.S., M.S., A.K., J.S., J.H., B.T., G.N., J.v.S., and M.N.
- Visualization: Y.-N.B.
- Supervision: A.K., G.N., M.N.
- Project administration: M.N.

All authors have read and agreed to the published version of the manuscript.

### Authors’ ORCIDs

- Yann-Nicolas Batzler: [0000-0002-4436-6448]
- Manuela Schallenburger: [0000-0002-3364-6137]
- Tabea Sammer: [0000-0003-4903-8109
- Jan Haussmann: [0000-0002-9315-269X]
- Balint Tamaskovics: [0000-0002-2533-0167]
- Stefanie Otten: [0009-0002-7064-6109]
- Corinna Fohler: [0009-0001-6626-7133]
- Jacqueline Schwartz: [0000-0002-0945-1292]
- André Karger: [0000-0002-4819-0144]
- Günter Niegisch: [0000-0001-6929-8691]
- Martin Neukirchen: [0000-0002-2287-7896]

### Informed consent and ethics

All subjects gave their informed consent for inclusion before they participated in the study. All actors have given their consent for images of them to be published.

The study was conducted in accordance with the Declaration of Helsinki and the Declaration of Geneva. The protocol was approved by the Ethics Committee of Heinrich-Heine-University Düsseldorf (reference number 2023-2652).

### Data availability 

The original contributions presented in the study are included in the article. The developed questionnaire for the evaluation can be found in attachment 1 . Further inquiries can be directed to the corresponding author.

### Funding

The development of the elective was funded by Qualitätsverbesserungsmittel (QVM) of the state of North-Rhine Westphalia (QVM 10/22). 

## Competing interests

The authors declare that they have no competing interests. 

## Supplementary Material

Questionnaire (only in German)

## Figures and Tables

**Table 1:**
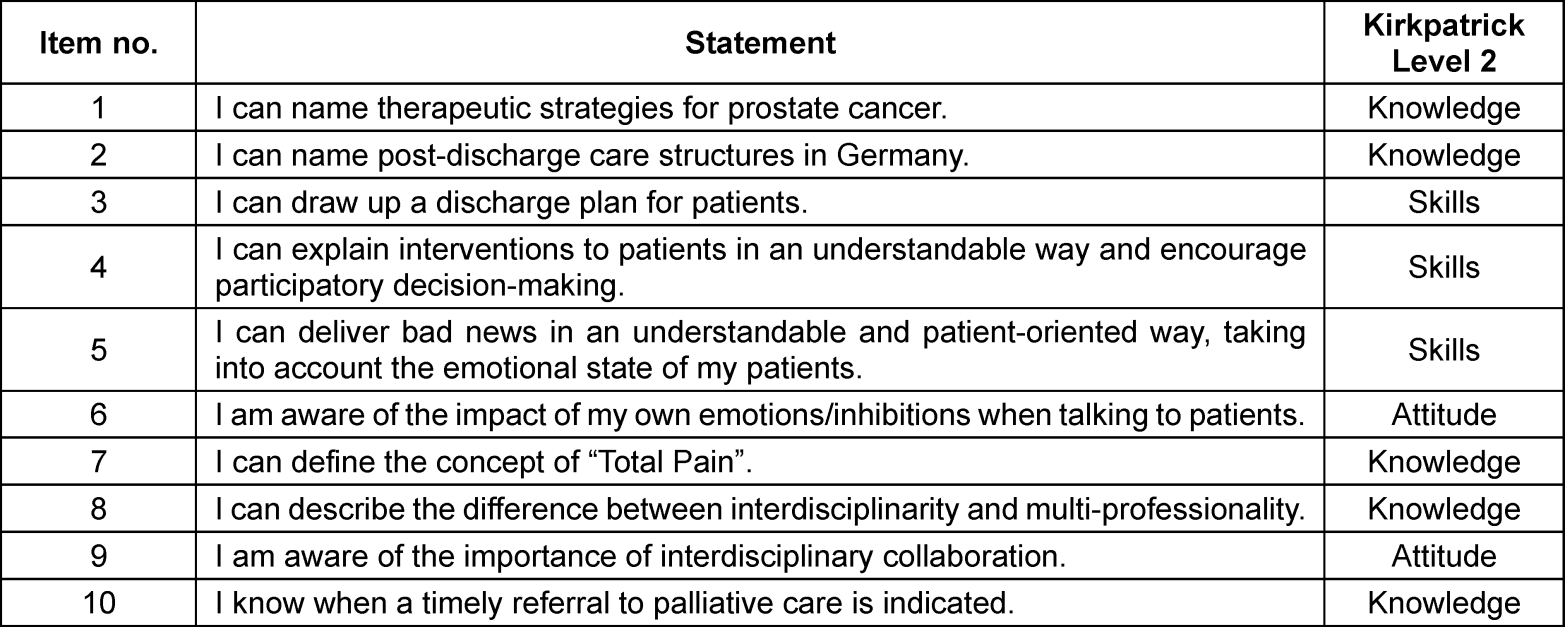
Items and their classification according to Kirkpatrick

**Table 2:**
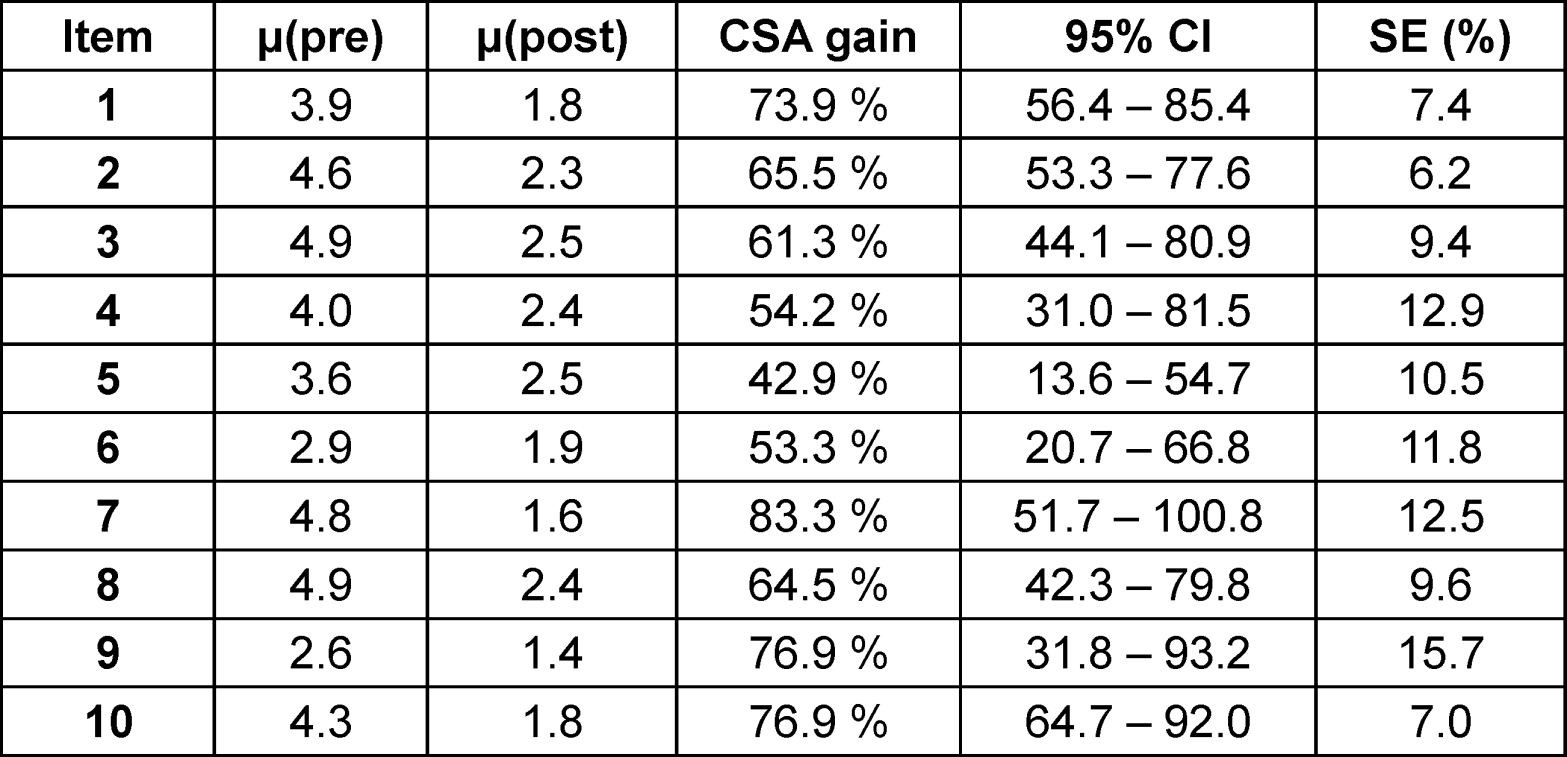
Items, CSA gains, confidence intervals, and standard errors
