# Supplementary material for: Promoting interdisciplinarity and the timely integration of palliative care through the development and implementation of a blended learning elective for medical students
Source: GMS J Med Educ. 2025 Apr 15;42(2):Doc24. doi: 10.3205/zma001748 (PMC12131507; doi:10.3205/zma001748)
Supplement: Questionnaire (only in German) [file JME-42-24-s-001.pdf]

Attachment 1: Questionnaire (only in German)

Teil 1: Evaluation

Die Struktur des Wahlfachs im Blockunterricht sagt mir zu.

Stimme überhaupt nicht zu	Stimme nicht zu	Weder noch	Stimme zu	Stimme voll und ganz zu.

Die Abfolge der Blöcke hat für mich Sinn ergeben.

Stimme überhaupt nicht zu	Stimme nicht zu	Weder noch	Stimme zu	Stimme voll und ganz zu.

Der Aufbau der einzelnen Blöcke hat für mich Sinn ergeben.

Stimme überhaupt nicht zu	Stimme nicht zu	Weder noch	Stimme zu	Stimme voll und ganz zu.

Die Gliederung des Wahlfachs in E-Learning, Seminaren, Demonstrationen und Simulationsgesprächen ist gelungen.

Stimme überhaupt nicht zu	Stimme nicht zu	Weder noch	Stimme zu	Stimme voll und ganz zu.

Die Inhalte des E-Learnings sind verständlich dargestellt.

**Stimme überhaupt nicht zu**	**Stimme nicht zu**	**Weder noch**	**Stimme zu**	**Stimme voll und ganz zu.**

Ich wünsche mir mehr e-Learning Inhalte.

**Stimme überhaupt nicht zu**	**Stimme nicht zu**	**Weder noch**	**Stimme zu**	**Stimme voll und ganz zu.**

Ich wünsche mir mehr Demonstrationen (OP, Strahlentherapie).

**Stimme überhaupt nicht zu**	**Stimme nicht zu**	**Weder noch**	**Stimme zu**	**Stimme voll und ganz zu.**

Ich wünsche mir mehr Simulationsgespräche.

**Stimme überhaupt nicht zu**	**Stimme nicht zu**	**Weder noch**	**Stimme zu**	**Stimme voll und ganz zu.**

Ich bin im Ganzen mit dem Wahlfach zufrieden.

**Stimme überhaupt nicht zu**	**Stimme nicht zu**	**Weder noch**	**Stimme zu**	**Stimme voll und ganz zu.**

Das Wahlfach vermittelt Inhalte, die für meine spätere Arbeit wichtig sind.

Stimme überhaupt nicht zu	Stimme nicht zu	Weder noch	Stimme zu	Stimme voll und ganz zu.

Die im Wahlfach vermittele Interdisziplinarität ist für meine spätere Arbeit wichtig.

Stimme überhaupt nicht zu	Stimme nicht zu	Weder noch	Stimme zu	Stimme voll und ganz zu.

Die im Wahlfach vermittelte Interdisziplinarität erlebe ich in dieser Form in vielen anderen Wahlfächern.

Stimme überhaupt nicht zu	Stimme nicht zu	Weder noch	Stimme zu	Stimme voll und ganz zu.

Ich würde das Wahlfach weiterempfehlen.

Stimme überhaupt nicht zu	Stimme nicht zu	Weder noch	Stimme zu	Stimme voll und ganz zu.

Die Inhalte entsprechen meinen Erwartungen bezüglich des Wahlfachs.

Stimme überhaupt nicht zu	Stimme nicht zu	Weder noch	Stimme zu	Stimme voll und ganz zu.

Attachment 1 to Batzler YN, Schallenburger M, Sammer T, Haussmann J, Tamaskovics B, Rehlinghaus M, von Schreitter J, Otten S, Fohler C, Schwartz J, Karger A, Niegisch G, Neunkirchen M. *Promoting interdisciplinarity and the timely integration of palliative care through the development and implementation of a blended learning elective for medical students.* GMS J Med Educ. 2024;42(2):Doc24. DOI: 10.3205/zma001748

Die Dozierenden sind ausführlich auf Fragen eingegangen.

Stimme überhaupt nicht zu	Stimme nicht zu	Weder noch	Stimme zu	Stimme voll und ganz zu.

Die Blöcke boten ausreichend Raum für Diskussion.

Stimme überhaupt nicht zu	Stimme nicht zu	Weder noch	Stimme zu	Stimme voll und ganz zu.

Die Lehrenden wirken kompetent.

Stimme überhaupt nicht zu	Stimme nicht zu	Weder noch	Stimme zu	Stimme voll und ganz zu.

Verbesserungsvorschläge (Freitext):

Attachment 1 to Batzler YN, Schallenburger M, Sammer T, Haussmann J, Tamaskovics B, Rehlinghaus M, von Schreitter J, Otten S, Fohler C, Schwartz J, Karger A, Niegisch G, Neunkirchen M. *Promoting interdisciplinarity and the timely integration of palliative care through the development and implementation of a blended learning elective for medical students.* GMS J Med Educ. 2024;42(2):Doc24. DOI: 10.3205/zma001748

Teil 2: Wissenszuwachs

Ich kann die Behandlungsstrategien des Prostatakarzinoms grob benennen.

	1	2	3	4	5	6
**Vor dem Wahlfach**						
**Nach dem Wahlfach**						

Ich kann poststationäre Versorgungsstrukturen in Deutschland beschreiben.

	1	2	3	4	5	6
**Vor dem Wahlfach**						
**Nach dem Wahlfach**						

Ich kann einen Entlassplan für Patient*innen erstellen.

	1	2	3	4	5	6
**Vor dem Wahlfach**						
**Nach dem Wahlfach**						

Ich kann Patient*innen verständlich über Interventionen aufklären und zur partizipativen Entscheidungsfindung ermutigen.

	1	2	3	4	5	6
**Vor dem Wahlfach**						
**Nach dem Wahlfach**						

Ich kann schlechte Nachrichten verständlich und patientenorientiert sowie unter Berücksichtigung der emotionalen Verfassung meiner Patient*innen übermitteln.

	1	2	3	4	5	6
**Vor dem Wahlfach**						
**Nach dem Wahlfach**						

Ich bin mir der Bedeutung meiner eigenen Emotionen/Hemmungen im Gespräch mit Patient*innen bewusst.

	1	2	3	4	5	6
**Vor dem Wahlfach**						
**Nach dem Wahlfach**						

Ich kenne das Total Pain Konzept.

	1	2	3	4	5	6
**Vor dem Wahlfach**						
**Nach dem Wahlfach**						

Ich kann den Unterschied zwischen Interdisziplinarität und Multiprofessionalität beschreiben.

	1	2	3	4	5	6
**Vor dem Wahlfach**						
**Nach dem Wahlfach**						

Ich weiß um die Bedeutung interdisziplinärer Zusammenarbeit für Patient*innen.

	1	2	3	4	5	6
**Vor dem Wahlfach**						
**Nach dem Wahlfach**						

Attachment 1 to Batzler YN, Schallenburger M, Sammer T, Haussmann J, Tamaskovics B, Rehlinghaus M, von Schreitter J, Otten S, Fohler C, Schwartz J, Karger A, Niegisch G, Neunkirchen M. *Promoting interdisciplinarity and the timely integration of palliative care through the development and implementation of a blended learning elective for medical students.* GMS J Med Educ. 2024;42(2):Doc24. DOI: 10.3205/zma001748

Ich weiß, wann eine zeitgerechte Anbindung an die Palliativmedizin indiziert ist.

	1	2	3	4	5	6
**Vor dem Wahlfach**						
**Nach dem Wahlfach**						

Attachment 1 to Batzler YN, Schallenburger M, Sammer T, Haussmann J, Tamaskovics B, Rehlinghaus M, von Schreitter J, Otten S, Fohler C, Schwartz J, Karger A, Niegisch G, Neunkirchen M. *Promoting interdisciplinarity and the timely integration of palliative care through the development and implementation of a blended learning elective for medical students.* GMS J Med Educ. 2024;42(2):Doc24. DOI: 10.3205/zma001748
